# Study of the Effects of Air Pollutants on Human Health Based on Baidu Indices of Disease Symptoms and Air Quality Monitoring Data in Beijing, China

**DOI:** 10.3390/ijerph16061014

**Published:** 2019-03-20

**Authors:** Shaobo Zhong, Zhichen Yu, Wei Zhu

**Affiliations:** 1 Beijing Research Center of Urban Systems Engineering, Beijing 100035, China; zhongshaobo@gmail.com; 2 Department of Engineering Physics, Tsinghua University, Beijing 100084, China; yuzhichen93@163.com

**Keywords:** air pollution, air quality index, generalized additive model, Baidu index, cardio- and cerebrovascular disease, respiratory disease

## Abstract

There is an increasing body of evidence showing the impact of air pollutants on human health such as on the respiratory, and cardio- and cerebrovascular systems. In China, as people begin to pay more attention to air quality, recent research focused on the quantitative assessment of the effects of air pollutants on human health. To assess the health effects of air pollutants and to construct an indicator placing emphasis on health impact, a generalized additive model was selected to assess the health burden caused by air pollution. We obtained Baidu indices (an evaluation indicator launched by Baidu Corporation to reflect the search popularity of keywords from its search engine) to assess daily query frequencies of 25 keywords considered associated with air pollution-related diseases. Moreover, we also calculated the daily concentrations of major air pollutants (including PM_10_, PM_2.5_, SO_2_, O_3_, NO_2_, and CO) and the daily air quality index (AQI) values, and three meteorological factors: daily mean wind level, daily mean air temperature, and daily mean relative humidity. These data cover the area of Beijing from 1 March 2015 to 30 April 2017. Through the analysis, we produced the relative risks (RRs) of the six main air pollutants for respiratory, and cardio- and cerebrovascular diseases. The results showed that O_3_ and NO_2_ have the highest health impact, followed by PM_10_ and PM_2.5_. The effects of any pollutant on cardiovascular diseases was consistently higher than on respiratory diseases. Furthermore, we evaluated the currently used AQI in China and proposed an RR-based index (health AQI, HAQI) that is intended for better indicating the effects of air pollutants on respiratory, and cardio- and cerebrovascular diseases than AQI. A higher Pearson correlation coefficient between HAQI and RR_Total_ than that between AQI and RR_Total_ endorsed our efforts.

## 1. Introduction

Respiratory, and cardio- and cerebrovascular diseases are generally considered associated with air pollution. According to some studies [1,2,3,4,5,6], short-term exposures are the main hazards that exacerbate symptoms or cause acute forms of diseases, while long-term exposures are probably the main hazards that cause this type of diseases. These studies show that the increase in air particulate matter, SO_2_, NO_2_, CO, O_3_, and other pollutants can worsen respiratory, and cardio- and cerebrovascular diseases [7,8,9,10,11,12,13,14,15]. An increasing body of evidence shows that air pollutants can significantly reduce lung functions and human body immune function and increase the prevalence of malignant tumors [16,17,18,19,20].

The acute health effects of short-term exposure to air pollutants were mainly studied using time-series studies, case-crossover studies, and panel studies [21]. There are some approaches to the assessment of health risk caused by air pollution. Risk-based approaches [22] and epidemiological approaches [23] are commonly applied. The risk-based approaches examine the way in which pollutants enter the body to estimate the exposure dose and risk. The routine epidemiological approaches evaluate the impact of exposure to air pollution based on the regressed relative risk (morbidity or mortality) with time series from hospital clinic data, pollutant concentration data, meteorological data, and other data sources. One of the widely used methods in time-series studies is based on the generalized additive model (GAM) with Poisson regression. Klot [24] found that an increase by 10 µg/m^3^ in the particulate matter smaller than 10 micrometers (PM_10_) led to a risk of 1.021 (95% confidence intervals (CIs): 1.004–1.039) in heart diseases such as acute myocardial infarction in cities throughout Europe from 1992 to 2000. Villeneuve [25] confirmed that, in April–September, the risk of acute ischemic stroke was 1.11 (95% CIs: 1.01–1.22) caused by the increase in SO_2_ concentration, and the risk of acute ischemic stroke caused by NO_2_ was 1.17 (95% CIs: 1.05–1.31) in Edmonton, Canada. Huang et al. [26] studied the relative risk (RR) values of cardiovascular and respiratory mortality caused by air pollution and visibility in Shanghai, China. In a recent systematic review and meta-regression analysis, Achilleos et al. found a 0.89% (95% CIs: 0.68, 1.10%) increase in all-cause, a 0.80% (95% CIs: 0.41, 1.20%) increase in cardiovascular, and a 1.10% (95% CIs: 0.59, 1.62%) increase in respiratory mortality per 10μg/m^3^ increase in the particulate matter smaller than 2.5 micrometers (PM_2.5_) [27].

Although the hospital outpatient records present detailed information such as age, sex, and disease history, thus representing good data sources for health impact analysis, these data have some disadvantages, including high difficulty of data acquisition, small sampling range, and uneven sampling. The Baidu index (BI) is an evaluation of keyword search popularity launched by Baidu Corporation (www.baidu.com). With a daily average search volume of up to six billion, the Baidu index has several advantages over hospital outpatient data: a wide range of sampling, uniform sampling, and easy to obtain. However, there is a basic assumption for using internet searches as surrogates of diseases, i.e., the search frequency simultaneously increases with cases in the study area increasing, even though there are some exceptional factors. For example, people may search for diseases or symptoms of friends or relatives that do not live in the search area or because they are only curious. Also, some people with disease will not make searches during symptoms. Under this assumption, the more significant the linear correlation is between increase in search volumes and increase in cases, the better the Baidu index is taken as a surrogate of disease occurrences. This assumption is not uncommon in some studies based on internet searches [28,29,30,31,32,33,34,35]. Reference [29] confirmed that an increase in Baidu index positively predicted the increase in HIV/AIDS (Human Immunodeficiency Virus that can cause Acquired Immune Deficiency Syndrome) incidence, even though the increase percentages were not different. Reference [32] showed that the Baidu index had a positive linear relationship with the local dengue fever occurrence. Reference [34] found a positive correlation between the volume of H7N9-related “cyber user awareness” and the epidemic situation. 

In this study, the Baidu index, deemed as representing symptom searches of respiratory, and cerebro- and cardiovascular diseases, was exploited in the place of hospital outpatient data to model the impact of air pollutants on human health. We also proposed a relative risk (RR)-based index that is supposed to better indicate the health risk of air pollution (specifically respiratory, and cerebro- and cardiovascular diseases), and compared it with China’s current air quality index (AQI) adopted by the Ministry of Environmental Protection. This new index can be used for health risk evaluation in our future mapping of the effect of air pollution on health in Beijing and the Chinese populations.

## 2. Methods

### 2.1. Study Area

This study was conducted in Beijing, the capital of China, where air pollution is severe. This area covers 16,410 km^2^, with a residential population of 21.7 million in 2016. The Gross Domestic Product (GDP) of the study area amounts to 3.35% of the whole country.

Unlike other cities where PM_2.5_ is high only in the winter, in Beijing, air pollution is high in both the autumn and winter [36]. According to the long-term observations performed by the Chinese Academy of Sciences [37], the average annual value of PM_2.5_ in Beijing was 92.7 μg/m^3^ during 2006–2017, reaching 110.7 μg/m^3^ in 2006, the historical maximum. Since 2006, the concentration of PM_2.5_ decreased by 3.36 μg/m^3^ each year. In 2013–2015, the concentration of PM_2.5_ in 74 cities in China showed a decreasing trend, and the concentration of PM_2.5_ in the Beijing–Tianjin–Hebei region overall decreased by 27.4%; however, the decrease was only 9.9% in Beijing. 

**Figure 1:**
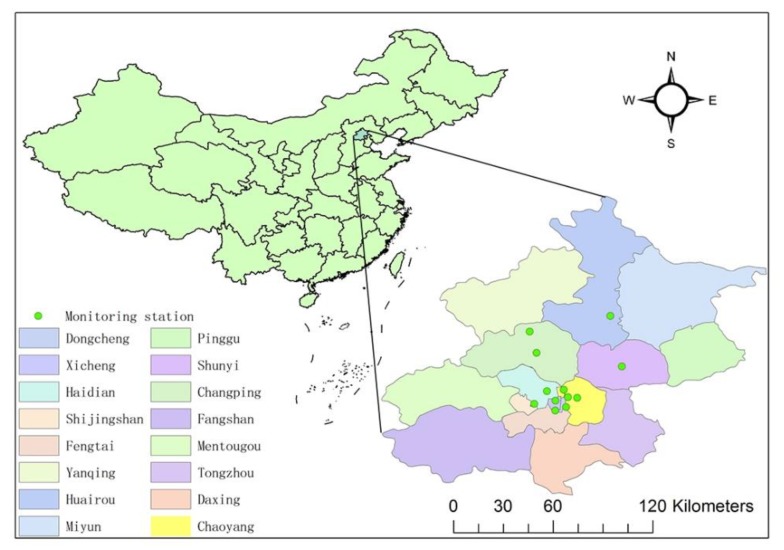
Locations of the study areas within China and the air quality monitoring stations.

There are 1436 monitoring stations of air quality in total all over China with data available from the Ministry of Environmental Protection of the People’s Republic of China (MEP) website (http://datacenter.mep.gov.cn/). Twelve of these stations are located in Beijing. The study area, its location in China, and the monitoring stations that provided pollutant data for our study are shown in Figure 1.

### 2.2. Data

There were three main types of data collected and preprocessed in our study: the AQI was considered as the causing factor, the Baidu index was taken as the effect factor, and meteorological observations were used as measures of condition factors.

#### 2.2.1. Meteorological Observations

We obtained hourly series of three meteorological factors (mean wind level, mean air temperature, and mean relative humidity) during 1 March 2015–30 April 2017 from meteorological stations located in Beijing City. These meteorological factors are presumed to have control over the effects of air pollution on health. The missing data were filled with interpolated values from their neighbor observations (if any) on the same day through linear interpolation.

#### 2.2.2. Air Quality Data

We downloaded hourly records of AQI and six individual pollutant concentrations: PM_10_, PM_2.5_, SO_2_, O_3_, NO_2_, and CO during 1 March 2015–30 April 2017 from the MEP website. The missing data were filled with interpolated values from their neighbor observations (if any) on the same day through linear interpolation.

According to the algorithm provided by the MEP Data Center, the AQI takes the maximum of the six individual air quality index (IAQI) values (SO_2_, NO_2_, PM_10_, PM_2.5_, O_3_, and CO). The IAQI was calculated as follows [38]:

(1) $$IAQI_{i}=\frac{IAQI_{Hi}-IAQI_{Li}}{BP_{Hi}-BP_{Li}}\left(C_{i}-BP_{Li}\right)+IAQI_{Li},$$

where *IAQI_i_* represents the individual air quality index of the *i*-th pollutant; *C_i_* is the concentration of the *i*-th pollutant; *BP_Hi_* and *BP_Li_* are the high and low values of the pollutant concentration limits closest to *C_i_*; and *IAQI_Hi_* and *IAQI_Li_* are the individual air quality indices corresponding to *BP_Hi_* and *BP_Li_*. Table 1, which is from the Chinese government’s Ambient Air Quality Standard (GB3095-2012) [39], can be used to look up the values of *IAQI_Hi_*, *IAQI_Li_*, *BP_Hi_*, and *BP_Li_*.

#### 2.2.3. Baidu Indices

Baidu is the largest search engine portal in China with a daily search volume up to six billion. The Baidu index is an evaluation indicator launched by Baidu Corporation (www.baidu.com) to reflect the search popularity of keywords from the search engine. It analyzes and calculates the weighted sum of the search times of keywords of interest by the network users from the Baidu Portal. Many researchers in China used Baidu index data in their studies [28,29,30,31,32,33,34,35,40]. Based on the results of the literature survey, we present 37 keywords that are assumed to be associated with air pollution-related diseases [17,18,19]. We obtained their Baidu indices from https://index.baidu.com/, where the search area was set to “Beijing all cities”, the time period was set to 1 March 1 2015–30 April 2017, and the type was the overall trend. As some of the keywords were not included in Baidu index, we ultimately obtained the Baidu indices for 25 keywords (Table 2). The Baidu index only includes the keywords that have a relatively high search volume, and 12 keywords (the English counterparts of the 12 Chinese keywords not included were ischemic heart disease, hypertensive heart disease, shortness of breath, myocardial disease, pericardial disease, stridor, whistle, acute and chronic rheumatic heart disease, cerebral atherosclerotic infarction, low-birth-weight baby, pulmonary failure, and other types of heart disease) were not included in the Baidu index, indicating that they are rarely entered due to very little use. These keywords are either statistically insignificantly correlated with short-term air pollution, or they have more commonly used alternatives in the 25 keywords. In summary, not including the 12 keywords had little effect on our study and was statistically negligible.

We divided the 25 keywords into two categories: “respiratory system” and “cardio- and cerebrovascular”, and then we added all the indices of each category together and obtained the respiratory total index and cardio- and cerebrovascular total index. 

### 2.3. Correlation Analysis

Pearson correlation and *coplot* were used to measure the association between air quality data and the Baidu search indices. Pearson correlation is the most commonly used statistic to depict the linear relationship between two variables [41], while *coplot* is an exploratory graphical method to investigate the relationship between a pair of variables (*Y*1 and *Y*2) conditioned on a third variable (*X*) [42,43]. Here, it was used in the exploration of how the relationship between AQIs and Baidu index varied across meteorological factors. 

#### 2.3.1. Pearson Correlation

For two variables, say, *X* and *Y*, which have observations [*x_n_*] and [*y_n_*], respectively, the Pearson correlation coefficient is defined as

(2) $$r_{X,Y}=\frac{Cov\left(X,\;Y\right)}{\sigma_{X}\sigma_{Y}}=\frac{E\left[\left(X-\mu_{X}\right)\left(Y-\mu_{Y}\right)\right]}{\sigma_{X}\sigma_{Y}},$$

where Cov(X,Y) represents the covariance of *X*,*Y*; $\mu_{X}$ and $\mu_{Y}$ represent the means of *X* and *Y*; *E*[·] expresses a mathematical expectation; and $\sigma_{X}\;\mathrm{and}\;\sigma_{Y}$ are the standard deviations of *X* and *Y*, respectively.

In practical use, the formula for calculating the Pearson correlation coefficient using the observations is

(3) $$r_{xy}=\frac{\sum x_{i}y_{i}-n\bar{x}\bar{y}}{\left(n-1\right)s_{x}s_{y}}=\frac{n\sum x_{i}y_{i}-\sum x_{i}\sum y_{i}}{\sqrt{n\sum x_{i}^{2}-{\left(\sum x_{i}\right)}^{2}}\sqrt{n\sum y_{i}^{2}-{\left(\sum y_{i}\right)}^{2}}},$$

where $\bar{x}\left(\bar{y}\right)$ and *s_x_*(*s_y_*) are the mean and variance of [*x_n_*]([*y_n_*]), respectively; *r_xy_* is in the range of [−1, 1].

#### 2.3.2. Coplot

A *coplot* is essentially a composition of multiple scatterplots of two variables *Y*1 and *Y*2 conditional on a third variable *X*. The observations of *Y*1 and *Y*2 are divided into multiple groups according to the value intervals of *X* and scatterplotted. The value intervals can overlap. There are identical numbers of observations for each scatterplot. Generally, the scatterplots are arranged in a matrix from left to right and from bottom to top, corresponding to the ordering of the value intervals, and there is an additional component that is called the “Given” panel, which shows the value intervals of *X*. Since each scatterplot has the same number of observation samples, the sampling errors are homogeneous for each scatterplot. Thus, conditional correlation analysis between two variables can be visualized clearly with a *coplot*.

### 2.4. Exposure Assessment

#### 2.4.1. Statistical Modeling

The impact of air pollution on health is complex (nonlinear) and there may be cross effects between different pollutants. Pollutant concentration is also closely associated with weather factors and time. Considering these situations, we selected a GAM approach to study the relationship between air pollution and the Baidu index. The GAM is a semiparametric expansion of the generalized linear model (GLM) [44], which assumes that the functions are additive and that the composition of the functions is smooth. The GAM can better analyze this relationship because it can use the nonparametric smooth spline functions to fit the curve flexibly [45,46,47,48]. The basic formula of GAM is as follows:

*G*(*E*(*Y*)) = Intercept + *f*_1_(*x*_1_) + … + *f_m_*(*x_m_*), (4)

where E(Y) is the expectation of the response variable *Y*, and *G*(•) is the link function, the selection of which depends on the probability distribution of the response variable. Gaussian distribution and Poisson distribution are the most commonly used link functions in real-world applications, while *f_i_*(*x_i_*), *i* = 1, 2, 3,…, *m* represents the smooth functions of the *m* explanatory variables. More complex forms of GAM models also incorporate additional linear variables or dummy variables. 

Considering the adjustment of meteorological factors for the effects of air pollution on morbidity as done in some studies [3,5,26,48,49], we incorporated wind, temperature, and humidity into the exploratory variables. For time-series observations, it is common practice to extract long-term trends and changes in the cycle of working days. Because daily search counts typically follow a Poisson distribution, a GAM with log link and Poisson error, combined with the basic assumption that an increase in symptoms of the concerned diseases leads to a simultaneous increase in internet searches, is expected to reasonably associate air quality with smooth fluctuations in daily morbidity. This treatment is also consistent with several other time-series studies [50,51,52]. We specified the following GAM model formula:

*Y_t_* ~ Poisson(*λ_t_*) log*λ_t_* = Intercept + βAQI*_t_* + DOW + WIND + S(*Time*,*k*_1_) + S(*Temp*,*k*_2_) + S(*Humi*,*k*_3_) (5)

where *Y_t_* denotes the individual or total Baidu index; AQI*_t_* represents the air quality index and β is the corresponding regression coefficient; S(•) represents the smoothing splines, while *k*_1_, *k*_2_, and *k*_3_ are the degrees of freedom of smoothing splines; *Time* is the calendar time; DOW is the day of the week representing the dummy variable of Monday to Sunday; WIND is the daily mean wind speed level (also a dummy variable); *Temp* is the daily mean temperature; and *Humi* is the daily mean relative humidity.

We mainly optimized the model from two aspects: (1) identification of time lags, and (2) removal of the indices with weak correlations. On one hand, although we were examining the short-time effects of air pollutants, the effects may not appear simultaneously, instead showing a lag effect. We used the AQI to represent the total condition of air pollution, and took RTI and CTI as the response variables. We changed the delay of the time series of the two indices, and found time lags with the highest value of β. On the other hand, respiratory, and cardio- and cerebrovascular diseases are generally inextricably linked to air pollution, but each sub-index cannot be significantly associated with air pollution. We took 25 sub-indices as the response variables, and we fit each one to the exploratory variables; then, we eliminated those indices with low correlations (judged by β, *R^2^*, and deviance explained). As a result, we recalculated RTI and CTI. 

In summary, we specified and fitted a GAM to obtain the estimated log-relative βs of AQI following the basic steps of GAM: (1) determining the explanatory variables, (2) determining the link function, (3) optimizing the model, and (4) evaluating the results.

We selected the open-source software R (x64 Ver3.4.0) to carry out the GAM analysis (mainly using the “mgcv” package) [53,54]. To facilitate the comparison with existing studies, the results were presented as the percent change in daily searches per 10 ug/m^3^ increase in AQI (or IAQIs).

#### 2.4.2. Relative Risk (RR)

In epidemiology, relative risk (RR) is expressed as the ratio of risk of the outcome in one group compared with another group. It is worth noting that the risk ratio is different from the odds ratio, even though the latter is often interpreted as if it were the risk ratio [55]. In this study, based on the exposure–response coefficient β obtained from the GAM model, we calculated the logarithm of relative risk change (LRR, the natural logarithm of the RR) when the pollutant concentration changed by one unit. The LRR was then used to quantitatively measure the risk. Furthermore, the inter-quartile range (IQR) of the pollutant concentration was defined as the unit concentration. According to the above definition, the calculation formula of RR was RR *=* exp(β × IQR); correspondingly, the 95% CIs of RR were calculated as exp((β ± 1.96 SE) × IQR) [56]. This implies that the percentage change in the Baidu index was (RR − 1) × 100% for an increase of one IQR unit in pollutant concentration. Therefore, when the pollutant concentration changed by 10%, the percentage of the change in Baidu index was ((10/IQR) × (RR − 1)) × 100%.

### 2.5. Health AQI

We assumed that there was a regressed RR for each pollutant according to the GAM. Referring to the work of References [57,58,59,60], the short-term total exposure risk of the day can be defined as

(RR − 1)_total_ = max((*c_i_*/IQR*_i_*) × (RR*_i_* − 1)), (6)

where *i* = 1, …, 6 (the number of pollutants under consideration), RR*_i_* and IQR*_i_* represent the relative risk and inter-quartile range for pollutant *I*, respectively, and *c_i_* is the corresponding day-averaged concentration.

For convenience, we defined a pollutant sub-index (PSI) to reflect the contribution of individual pollutants to the overall risk.

PSI*_j_* = *c_j_* × *a_j_*, (7)

where the subscript *j* refers to the *j*-th pollutant, *c_j_* refers to the corresponding day-averaged concentration, and *a_j_* is directly proportional to the incremental risk values (RR*_i_* − 1). We then defined a new AQI as

HAQI = max(PSI*_j_*). (8)

This new AQI focuses on effects of air pollution on health; thus, we called it the health AQI (HAQI).

## 3. Results

### 3.1. Data Exploration

**Figure 2:**
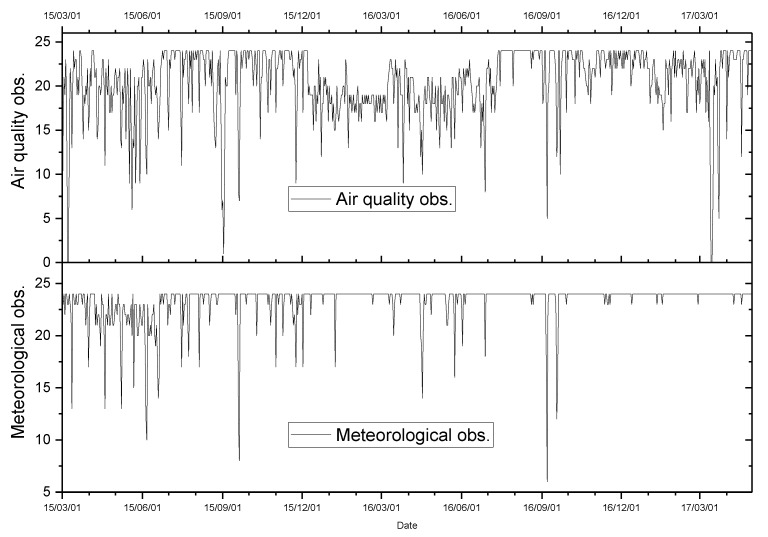
The number of daily observations during the study period.

**Figure 3:**
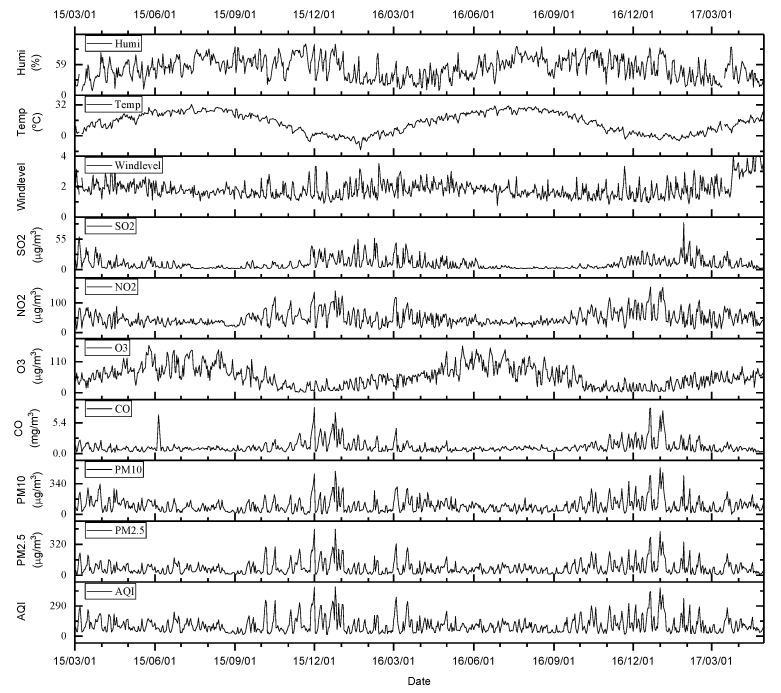
The time series of daily meteorological factors (mean temperature, mean relative humidity, and mean wind level), pollutant concentrations, and the Ministry of Environmental Protection (MEP) air quality index (AQI).

**Figure 4:**
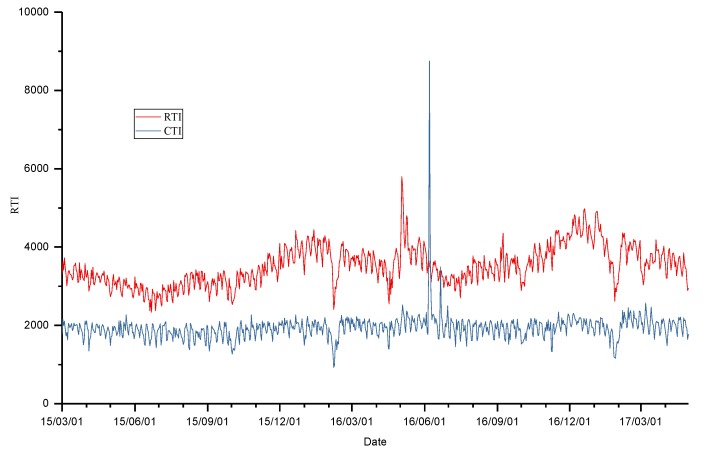
The time series of daily respiratory total index (RTI) and cardio- and cerebrovascular total index (CTI).

The hourly meteorological observations and pollutant concentration records from 1 March 2015, 12:00 a.m. to 30 April 2017, 11:00 p.m. were obtained. The number of missing data points for hourly air quality indices and meteorological observations was 535 (accounting for 2.8%) and 2819 (accounting for 14.8%). Figure 2 shows the numbers of valid observations every day during the study period. It is shown that AQIs had at least six records on any day for all days, and meteorological observations had no records for a few days (four days). Figure 3 shows the daily series of air quality indices and meteorological factors. During the study period, there was a summation of 4,314,272 Baidu indices of the selected 25 keywords. The RTI was 2,781,456 and the CTI was 1,532,816. There were about 5447 Baidu indices per day on average during the 792 days. Approximately, the RTI accounted for 64.5%. Figure 4 shows the daily series of RTI and CTI. As seen, there were obvious outliers in the beginning of June 2016. After checking the original data, we found that the outliers were during 6–11 June 2016. Since we did not know what caused these outliers, we excluded these outliers. We linearly interpolated these hourly data for missing points on the same day, and then integrated them into daily series through averaging the observations and interpolated values of all days. If there were no observations in a day (12:00 a.m. to 11:00 p.m.), the day was marked as having no data. 

Table 3 shows the summary statistics of the obtained daily AQIs and meteorological observations. We dropped the data of the four days and finally took the time series with 788 daily data points to build the GAM. 

**Figure 5:**
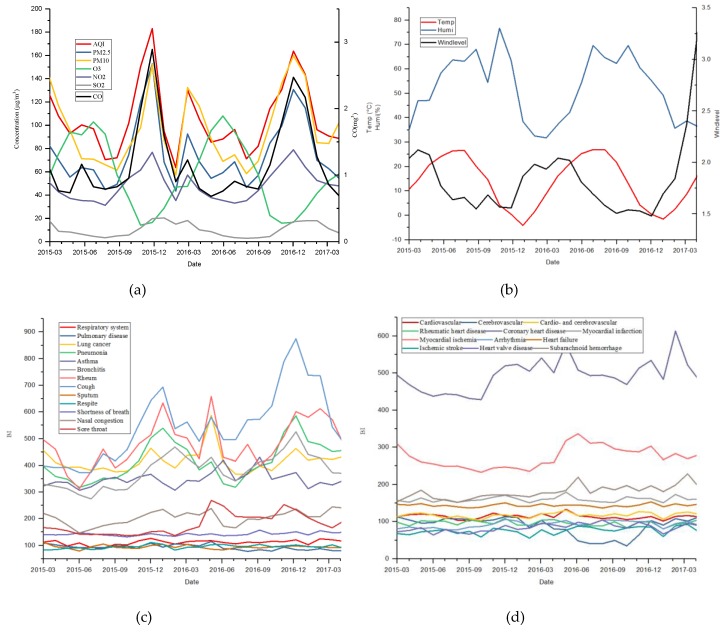
(**a**) AQI and six major air pollutants, where CO is shown on the right side of the vertical axis; (**b**) meteorological factors, where wind level is shown on the right side of the vertical axis; (**c**) respiratory-related Baidu indices; and (**d**) cardio- and cerebrovascular-related Baidu indices.

The monthly total search index of 25 selected keywords, the monthly AQI and six pollutant concentrations, and the monthly meteorological observations (obtained through averaging the daily data) are plotted in Figure 5. Figure 5a shows that almost all the trends of AQIs were similar except for O_3_. The curves approximately indicate high values in the winter and low values in the summer (except for O_3_, which had the inverse change). Figure 5b shows that temperature, relative humidity, and wind level had trends with one-year cycles. Figure 5c shows the respiratory-related search indices and indicates that all curves could be clearly divided into two groups. Among them, “bronchitis”, “asthma”, “lung cancer”, “pneumonia”, “rheum”, and “cough” had a higher search volume, and almost all of their curves had peaks in the winter and valleys in the summer except asthma, which had no clear cycle. Figure 5d shows the cardio- and cerebrovascular-related search indices. “Coronary” and “myocardial” had the highest search volume.

From the Pearson correlation coefficients shown in Table 4, the air quality indices and RTI had relatively high values of *r*, and the corresponding *p*-values were less than 0.01, which indicates significant correlation. In contrast, a correlation between pollution indices and CTI was not obvious in terms of the values of *r* and *p*-values. In all air quality indices, O_3_, NO_2_, CO, and RTI had the highest correlations. It is worth noting that O_3_ had a significant negative correlation with RTI.

**Figure 6:**
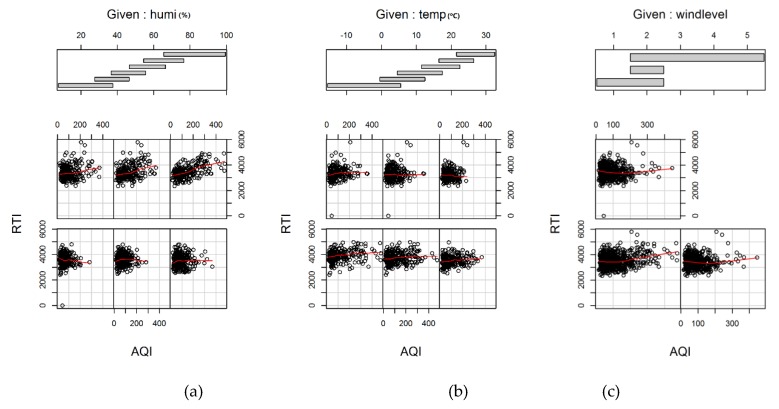
Coplot showing the relationship between the variables air quality index (AQI) and RTI conditioned on meteorological factors (humidity, temperature, and wind level): (**a**) the relationship between RTI and AQI conditioned on humidity; (**b**) the relationship between RTI and AQI conditioned on temperature; and (**c**) the relationship between RTI and AQI conditioned on wind speed.

Figure 6 shows scatter plots of AQI and RTI conditional on several meteorological factors, showing which curves were fitted to indicate the trends more clearly. The individual panels should be viewed from left to right, and bottom to top. Taking Figure 6c as an example, the lower left is the AQI and RTI scatter plot corresponding to the wind level ranging from 0.5–2.5, while the lower right is that from 1.5–2.5, and the upper left is that from 1.5–5.5. Meteorological factors were segmented based on the same number of samples per segment. Figure 6a shows that, when there is a higher humidity (*Humi*), RTI increases with AQI increasing. Figure 6b shows that the lower the temperature (*Temp*) is, the greater the impact is of AQI on the RTI. Figure 6c shows that the smaller the wind level is, the greater the impact is of AQI on the RTI. Synthetically, *Humi* had the greatest impact on AQI and RTI.

According to the coplot graphs, the same air quality index had a much higher correlation with RTI than with CTI given the same meteorological factors. The results were consistent with the results of the Pearson correlation coefficient. In addition, all groups generally showed similar trends: (1) when *Humi* increased, RTI increased faster with AQI increasing; (2) the lower the temperature was, the clearer the impact was of AQI on RTI; and (3) the smaller the wind level was, the greater the impact was of AQI on RTI. Among the three meteorological factors, the impact of *Humi* was the greatest.

### 3.2. Health Impact Evaluation

Because time itself has a high correlation with air quality, the time smoothing functions with a high degree of freedom are sensitive to the short-term air quality changes and may lead to overfitting. In order to explore the long-term trend of effects of time on Baidu indices, in this study, we confined the degree of freedom of time smoothing functions to 1–4, as commonly proposed in some studies [26,48,50,52]. Through graphically analyzing the time smoothing functions with those different degrees of freedom, we found that the time smoothing function with three degrees of freedom had the best consistency with the observation series. Furthermore, based on published literature [4,61], three degrees of freedom (whole period of study) for mean air temperature and mean relative humidity could control well for the meteorological effects on mortality and, thus, it was chosen to be used in our models. In summary, we finally took *k*_1_ = *k*_2_ = *k*_3_ = 3 in Equation (3) to obtain the estimated log-relative rate β.

Many researchers found that the health effects of air pollution on respiratory and cardiovascular diseases have a hysteresis of 0–6-day lags [25,48]. Therefore, we delayed the time series of the RTI and CTI and then carried out GAM regression analysis. The results are shown in Table 5. We can see that the βs of AQI varied significantly with the different lag periods. The regression coefficients reached a maximum when the time lag was three days. This lag is also consistent with the results obtained by other studies through hospital cases [25,48]. Therefore, the GAM analysis was performed with a time lag of three days.

Comparing the results of GAM regressing the RTI and CTI on the AQI, we can see that the *R^2^* and the explained level of the RTI were much higher than those of the CTI, indicating that the effects of air pollution on respiratory diseases are more significant than those on cardio- and cerebrovascular diseases. Air pollution, in contrast, can only statistically explain a lesser part of the change in cardio- and cerebrovascular incidences.

We also regressed the sub-indices with GAM and the results are shown in Table 6. We observed the regression curves of the AQI, meteorological factors, and time, and compared the contributions between them, so as to determine whether to retain the index. Eventually, 13 indices were retained, including seven respiratory indices, and six cardio- and cerebrovascular indices. Specifically, when *β* was larger than that of the total index, meaning that the influence of the sub-index was significant, the sub-index was retained. When *β* was small, and the *R^2^* and explained deviance was also small, it meant that, although the impact of the sub-index was smaller, it was more significant compared with meteorological factors and time and, thus, the sub-index should also be retained. The sub-index was removed when *β* was small, and the *R^2^* and explained deviance were less than 0.1, indicating that AQI, meteorological factors, and time were not significant. The sub-index was removed when *β* was small, and *R^2^* and explained deviance were high, indicating that meteorological factors and time were more significant than the sub-index.

### 3.3. RR of Air Pollutants

Taking RTI and CTI as the response variables, and the concentrations of the six pollutants as explanatory variables, and considering meteorological factors and time, we carried out a GAM Poisson regression analysis with three-day lag. The *p*-values of the explanatory variables for each model were all less than 0.001, indicating significant contributions of these pollutants to total indices. The results are shown in Table 7, indicating that the RR values of the six pollutants for cardio- and cerebrovascular diseases were higher than those for respiratory diseases. However, the differences were not great; NO_2_ and O_3_ had the highest RR values, followed by PM_10_ and PM_2.5_.

### 3.4. Performance of HAQI

Next, we compared HAQI with AQI in terms of their capability of indicating health effects. We took PM_2.5_ as a benchmark and selected the closest limit of 500 μg/m^3^ (the maximum daily concentration of PM_2.5_ during the study period was 478 μg/m^3^); then, IAQI = PSI = 500, RR_Total_ = (500/IQR_PM2.5_) × (RR_PM2.5_ − 1) + 1 = 1.223. Thus, we further established the relationship between RR_Total_ and IAQI and PSI. If pollutants have the same RR_total_ values at different concentrations, they have the same PSI values. The pollutant concentrations corresponding to different PSI values could be calculated through linearly interpolating RR_total_. The concentration values of the six pollutants corresponding to PSI and RR_Total_ were calculated and they are shown in Table 8. It is worth noting that the breakpoints of PSI and RR_Total_ were somehow a little arbitrary. We calculated pollutant concentrations at evenly spaced breakpoints for PSI and RR_Total_. Since the concentrations were calculated based on relative risk of individual pollutants, they should be different from the currently used AQI that reflects the comprehensive effects of air pollution on environment, ecology, and buildings, as opposed to health.

**Figure 7:**
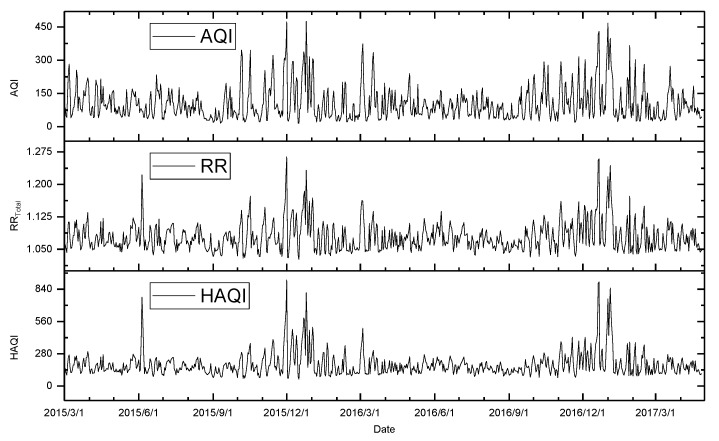
Curves of AQI, HAQI, and relative risk (RR_Total_) according to their daily data during the study period.

To evaluate the effects of the currently used AQI and the HAQI in expressing health outcome, we plotted the three curves of AQI, HAQI, and the daily exposure risk RR_Total_, as shown in Figure 7, showing that the overall changes of the three curves were similar, but there were obvious differences in local places. For quantitative comparison, the Pearson correlations between AQI, HAQI, and RR were calculated. The correlation coefficient between AQI and RR was 0.86, with a *p*-value < 0.001. The correlation coefficient between HAQI and RR was 0.95, with a *p*-value <0.001. In this sense, the HAQI was a little better for representing the short-term risk of air pollution. 

## 4. Discussion

In this paper, we focused on the evaluation of the short-term effect of air pollution on human health. An assumption was made that the internet searches of keywords of air pollution-related diseases was positively correlated with some symptoms of diseases including respiratory, and cardio- and cerebrovascular ones. The search data were used as indicators of the disease outcome instead of hospital outpatient data. These search data are supposed to avoid some disadvantages from hospital outpatient data such as high difficulty in data acquisition, small sampling range, and uneven sampling. 

A GAM was employed to model the association between internet search and air pollution. Specifically, a log form of response variable with Poisson distribution was used, and we improved the model mainly in two aspects: (1) the time lag was explored and incorporated into the regression form, and (2) some non-significant indices were eliminated according to several evaluation indices (*r*, *R^2^*, *p*-value, and deviance explained). Through the analysis, we obtained the RRs of the six air pollutants for respiratory, and cardio- and cerebrovascular diseases. The results show that the risk of O_3_ and NO_2_ for all the concerned diseases in this study was higher than other pollutants. The risk of a certain pollutant was higher for cardio- and cerebrovascular diseases than for respiratory diseases. Furthermore, we proposed a RR-based health air quality index (HAQI), which is intended to provide an indicator for assessing the impact of air pollutants on human health. The comparison between the currently used Chinese AQI and the HAQI was made, showing HAQI to be a little better than AQI.

Pearson correlation coefficients between RTI and every air quality index except O_3_ showed significant positive correlations. O_3_ had a significant negative correlation with RTI, which can be easily confirmed from the curves of their daily series in Figure 2 and Figure 3, since the curves of RTI and O_3_ had approximately opposite changes. However, we found that the RR of O_3_ for RTI was still greater than 1 when we examined the RRs of individual air pollutants. Pearson correlation only calculates *r* between two variables regardless of the effect of other variables; as a result, it cannot accurately depict a multi-variate relationship. In fact, when we regressed RTI on O_3_ and took meteorological factors as control variables, the adverse effect of O_3_ on health was presented.

The Baidu index only represents samples from the population since it is calculated according to the overall searches from regional netizens. It may have a bias because of some exceptional searching behaviors. It also cannot differentiate according to different age groups and sexes and, therefore, it is difficult to establish a direct link between the health loss and the index. We were, therefore, unable to evaluate the health loss in detail, which is useful for health risk assessment. On the other hand, our study required the keywords used for estimating search volume of the concerned diseases: respiratory and cerebro- and cardiovascular diseases were reasonably selected. We assured this as much as possible by duly and carefully picking the most frequently used Chinese words in depicting symptoms of these diseases through a literature survey. Nonetheless, our study was a statistical analysis of several sets of time series (air pollution, weather, and morbidity represented by internet search frequency); the qualitative and quantitative relationships between them were only data-driven and cannot be pronouncedly confirmed as causal evidences.

Some studies reported little or no acute effects of air pollution on cerebrovascular diseases, whereas others showed that the acute effects of air pollution caused myocardial infarction, ischemic stroke, ischemic heart disease, and cardio- and cerebrovascular diseases, resulting in an increase in emergencies, outpatient intake, and death [62]. The reasons for the differences in these findings may be (1) cardio- and cerebrovascular diseases are a large class of diseases and air pollution may be associated with the morbidity of certain subtypes but not with another subclass of diseases, and (2) when these diseases are summed up to the major categories of cardio- and cerebrovascular disease, it may lead to no statistically significant result.

The RR values of respiratory, and cardio- and cerebrovascular diseases associated with NO_2_, SO_2_, and O_3_ in our study are both larger than those reported on health impact assessment in the WHO European region [63]. However, the RR values associated with PM_2.5_ and PM_10_ are smaller than those reported by the WHO (PM_2.5_ and PM_10_) and several studies of air pollution in Europe [3,64] (PM_10_). We also compared the results with three studies for cities in China [65,66,67]. The first investigated the associations between ambient air pollution and adult respiratory mortality in 32 major Chinese cities, and the RR values of respiratory diseases associated with PM_10_ were greater than ours. The second and third studies performed a nationwide analysis of associations between PM_2.5_ and SO_2_ concentrations and daily cause-specific mortality in 272 Chinese cities respectively, indicating that the RR values of cardiovascular and respiratory diseases associated with SO_2_ and PM_2.5_ were also greater than ours. A relative overall survey of effects of air pollution on health in Chinese populations was made by Reference [68], which also showed RR values of respiratory and cardiovascular diseases associated with NO_2_ and PM_10_ higher than ours, while those associated with O_3_ and SO_2_ were lower than ours. The RRs in those studies were derived from medical records of mortality or morbidity, whereas our study was based on an internet search of keywords representing disease symptoms, which may be quite different from each other. Next, because of the different physical characteristics of each person, the threshold of response to physical abnormalities is also different, along with their internet habits. Furthermore, people living in different regional environments (e.g., high-pollution environments like some Chinese cities) may have different response characteristics. As a result, a simple comparison between the results of this study and those of other studies is unreasonable. The benefit of our study is its incorporation of those who feel physical discomfort and mild symptoms, but choose not to seek medical advice. Our analysis is not subject to the specific criteria for morbidity or death and, thus, it was intended to provide a broader assessment of the risk of air pollution.

In our next study, we plan to obtain a more complete table of air pollution-related keywords through network opinion analysis and big data mining or to investigate the search intention in case of air pollution through a questionnaire survey. These data will be incorporated into the analysis process (e.g., by weighting the keywords). While it may be true that there are some disadvantages, hospital data do directly measure health, unlike the Baidu index. Internet search data and outpatient data are two important data sources for studying the impact of air pollutants on diseases and we should make comparisons between them and take their advantages together to get insight into the relationship between environment and diseases. For example, we can evaluate the validity of using the Baidu index to indicate the presence of disease through exploring the linear correlation between Baidu index and contemporaneous outpatient data. This will also be included in our future studies on inter-validation of the Baidu index and outpatient data as predictive of actual health outcomes.

## Figures and Tables

**Table 1:** Breakpoints for the individual air quality index (IAQI) according to the standard of the Ministry of Environmental Protection (MEP).

IAQI	SO_2_ (μg/m^3^) 24 h	NO_2_ (μg/m^3^) 24 h	PM_10_ (μg/m^3^) 24 h	CO (mg/m^3^) 24 h	O_3_ (μg/m^3^) 8 h	PM_2.5_ (μg/m^3^) 24 h
0	0	0	0	0	0	0
50	50	40	50	2	100	35
100	150	80	150	4	160	75
150	475	180	250	14	215	115
200	800	280	350	24	265	150
300	1600	565	420	36	800	250
400	2100	750	500	48	1000	350
500	2620	940	600	60	1200	500

**Table 2:** The selected keywords for the Baidu indices.

Category	Keywords
RTI (respiratory total index)	respiratory system, pulmonary disease, lung cancer, pneumonia, asthma, bronchitis, rheum, cough, sputum, respite, shortness of breath, nasal, congestion, sore throat
CTI (cardio- and cerebrovascular total index)	cardiovascular, cerebrovascular, cardio- and cerebrovascular, rheumatic, heart disease, coronary heart disease, myocardial infarction, myocardial, ischemia, arrhythmia, heart failure, ischemic stroke, heart valve disease, subarachnoid hemorrhage

**Table 3:** Summary statistics of air pollution data, meteorological observations, and Baidu searches in Beijing (1 March 2015–30 April 2017).

Data	Days	Mean ± SE	Min	P25	Median	P75	Max	IQR
Air pollution								
AQI	792	105.5 ± 2.72	15.0	50.5	83.7	135.9	475.2	85.4
PM_2.5_ (μg/m^3^)	792	74.8 ± 2.45	6.7	26.9	54.2	98.6	477.5	71.7
PM_10_ (μg/m^3^)	792	97.1 ± 2.70	0.0	41.6	79.7	129.2	518.3	87.6
CO (mg/m^3^)	792	1.2 ± 0.038	0.23	0.59	0.89	1.32	8.14	0.73
O_3_ (μg/m^3^)	792	57.5 ± 1.31	2.1	28.9	53.3	79.5	168.0	50.6
NO_2_ (μg/m^3^)	792	48.5 ± 0.87	10.4	31.6	42.8	59.8	153.5	28.2
SO_2_ (μg/m^3^)	792	10.3 ± 0.37	1.8	3.1	6.4	14.0	85.2	10.9
Meteorological observations								
^a^ Wind level	788	1.84 ± 0.021	0.75	1.43	1.72	2.09	4.67	0.66
Temperature (°C)	788	13.5 ± 0.37	-14.5	3.6	14.9	23.1	32.4	19.5
Relative humidity (%)	788	52.1 ± 0.73	8.0	35.8	52.7	68.5	98.6	32.7
Baidu indices								
RTI	792	3511.9 ± 17.79	2336.0	3144.0	3460.5	3837.5	5800.0	693.5
CTI	792	1935.4 ± 12.30	928.0	1792.5	1948.5	2071.0	8750.0	278.5

^a^ Wind level was determined according to the wind speed ranges defined in the standard “Wind Power Level” issued by the Chinese Meteorological Administration.

**Table 4:** The results of the Pearson correlation analysis (RTI: respiratory total index, CTI: cardio- and cerebrovascular total index).

Pollutant	RTI		CTI	
	*r* (95% CIs)	*p*−value	*r* (95% CIs)	*p*-value
AQI	0.21 (0.14,0.28)	9.84 ×10^−10^	0.05(−0.02, 0.12)	0.1288
PM_2.5_	0.23 (0.16,0.30)	1.57 ×10^−11^	0.05(−0.02, 0.12)	0.1781
PM_10_	0.23 (0.16, 0.29)	3.212 ×10^−11^	0.07(−0.00, 0.14)	0.0589
CO	0.33 (0.26,0.39)	<2.2 ×10^−16^	0.05 (−0.02, 0.12)	0.1683
O_3_	−0.40(−0.45, −0.33)	<2.2 ×10^−16^	0.07 (0.00,0.14)	0.03644
NO_2_	0.35 (0.29,0.41)	<2.2 ×10^−16^	0.04 (−0.03, 0.11)	0.2233
SO_2_	0.28 (0.21,0.34)	1.485 ×10^−15^	0.08 (0.01, 0.14)	0.03383

**Table 5:** The results with the generalized additive model (GAM) taking into account different time lags. Lags of 0–5 days were considered and the corresponding βs for RTI and CTI were calculated.

Lag (Day)	0	1	2	3	4	5
β for RTI (10^−4^)	2.178	2.489	2.835	3.05	3.073	2.468
β for CTI (10^−4^)	1.151	1.867	2.496	3.157	2.991	2.408

**Table 6:** Results with GAM considering a three-day lag, with AQI (and IAQI) as explanatory variables and Baidu indices (BIs) as response variables. The indices marked by “-“ indicate insignificance and were removed; the number of asterisks indicates how well the index is explained by AQI (IAQI).

BI	β	*R^2^*	Deviance Explained	Reserved
respiratory system	3.204 ×10^−4^	0.0845	8.27	*
pulmonary disease	5.437 ×10^−4^	0.0847	8.04	**
lung cancer	6.017 ×10^−4^	0.267	30.8	***
pneumonia	3.385 ×10^−4^	0.694	71	-
asthma	0.773 ×10^−4^	0.226	26	-
bronchitis	3.181 ×10^−4^	0.631	65.1	*
rheum	3.350 ×10^−4^	0.386	42.7	*
cough	1.942 ×10^−4^	0.737	75.8	-
sputum	0.142 ×10^−4^	0.037	5.06	-
respite	−3.04 ×10^−4^	0.0197	3.2	-
shortness of breath	0.819 ×10^−4^	0.0863	9.6	-
nasal congestion	3.927 ×10^−4^	0.384	40	***
sore throat	1.618 ×10^−4^	0.318	33.2	*
cardiovascular	3.277 ×10^−4^	0.11	10.2	**
cerebrovascular	4.877 ×10^−4^	0.216	19.7	**
cardio- and cerebrovascular	1.621 ×10^−4^	0.0975	9.37	-
rheumatic heart disease	0.932 ×10^−4^	0.0432	5.05	-
coronary heart disease	2.844 ×10^−4^	0.438	45.8	*
myocardial infarction	0.43 ×10^−4^	0.105	12.4	-
myocardial ischemia	1.332 ×10^−4^	0.285	30	-
arrhythmia	0.368 ×10^−4^	0.0883	9.96	-
heart failure	1.8 ×10^−4^	0.0815	9.1	*
ischemic stroke	−2.56 ×10^−4^	0.0765	7.09	-
heart valve disease	5.455 ×10^−4^	0.109	10.1	**
subarachnoid hemorrhage	6.747 ×10^−4^	0.0179	13.6	**

**Table 7:** Exposure–response assessment of the six pollutants. While the unit of CO concentration is mg/m^3^, the units of the other pollutant concentrations are μg/m^3^. The fifth column shows relative risks when pollutant concentrations change by one unit of IQR. The sixth column shows the increases of relative risks of the health outcome per 10 μg/m^3^ (per 1 mg/m^3^ for CO) increase in pollutant concentrations.

Total Index	Pollutant	IQR	β	RR (95% CIs)	(RR − 1) × 100% (95% CIs)
RTI	PM_2.5_	71.7	0.00044	1.0317 (1.0297–1.0338)	0.45% (0.42%–0.48%)
	PM_10_	87.6	0.000389	1.0353 (1.0332–1.0374)	0.40% (0.37%–0.42%)
	CO	0.7	0.0320	1.0227 (1.0212–1.0241)	3.24% (3.03%–3.44%)
	O_3_	50.6	0.000721	1.0375 (1.0333–1.0417)	0.73% (0.65%–0.82%)
	NO_2_	28.2	0.00135	1.0388 (1.0362–1.0414)	1.37% (1.28%–1.46%)
	SO_2_	10.9	0.00141	1.0156 (1.0135–1.0176)	1.42% (1.23%–1.61%)
CTI	PM_2.5_	71.7	0.000522	1.0378 (1.0351–1.0405)	0.53% (0.49%–0.57%)
	PM_10_	87.6	0.000393	1.0356 (1.0329–1.0384)	0.40% (0.37%–0.43%)
	CO	0.7	0.0373	1.0265 (1.0246–1.0284)	3.78% (3.51%–4.05%)
	O_3_	50.6	0.00197	1.1056 (1.0999–1.1113)	2.07% (1.96%–2.18%)
	NO_2_	28.2	0.00135	1.0390 (1.0355–1.0424)	1.38% (1.26%–1.50%)
	SO_2_	10.9	0.00312	1.0349 (1.0322–1.0376)	3.17% (2.93%–3.41%)

**Table 8:** The calculated concentrations for the six pollutants at evenly spaced breakpoints of pollutant sub-index (PSI) ranging from 0 to 500, given that PSI = 500 corresponds to the PM_2.5_ concentration of 500. a*_j_* was calculated according to Equation (7), which was used to calculate daily health AQI (HAQI) with Equation (8).

RR_Total_	PSI	SO_2_ (μg/m^3^) 24 h	NO_2_ (μg/m^3^) 24 h	PM_10_ (μg/m^3^) 24 h	CO (mg/m^3^) 24 h	O_3_ (μg/m^3^) 8 h	PM_2.5_ (μg/m^3^) 24 h
1.0000	0	0	0	0	0	0	0
1.0223	50	15.74	16.25	56.40	0.44	30.36	50
1.0446	100	31.48	32.50	112.80	0.89	60.71	100
1.0670	150	47.22	48.75	169.20	1.33	91.07	150
1.0893	200	62.95	65.00	225.60	1.77	121.42	200
1.1116	250	78.69	81.26	282.00	2.21	151.78	250
1.1339	300	94.43	97.51	338.40	2.66	182.13	300
1.1562	350	110.17	113.76	394.80	3.10	212.49	350
1.1786	400	125.91	130.01	451.21	3.54	242.84	400
1.2009	450	141.65	146.26	507.61	3.98	273.20	450
1.2232	500	157.38	162.51	564.01	4.43	303.55	500
a*_j_*		3.18	3.08	0.89	112.97	1.65	1.00

## References

[1] Schwartz J. (1996). Air pollution and hospital admissions for respiratory disease. Epidemiology.

[2] Braunfahrländer C., Ackermannliebrich U., Schwartz J., Gnehm H.P., Rutishauser M., Wanner H.U. (1992). Air pollution and respiratory symptoms in preschool children. Am. Rev. Respir. Dis..

[3] Atkinson R.W., Ross Anderson H., Sunyer J., Ayres J.O., Baccini M., Vonk J.M., Boumghar A., Forastiere F., Forsberg B., Touloumi G. (2001). Acute effects of particulate air pollution on respiratory admissions: Results from APHEA 2 project. Air Pollution and Health: A European Approach. Am. J. Respir. Crit. Care Med..

[4] Dominici F., Peng R.D., Bell M.L., Pham L., Mcdermott A., Zeger S.L., Samet J.M. (2006). Fine Particulate Air Pollution and Hospital Admission for Cardiovascular and Respiratory Diseases. JAMA J. Am. Med Assoc..

[5] Kowalska M. (2012). Short-Term Effect of Changes in Fine Particulate Matter Concentrations in Ambient Air to Daily Cardio-Respiratory Mortality in Inhabitants of Urban-Industrial Agglomeration (Katowice Agglomeration), Poland. Air Quality—New Perspective.

[6] Dockery D.W., Rd P.C. (1994). Acute respiratory effects of particulate air pollution. Annu. Rev. Public Health.

[7] Estévez-García J.A., Rojas-Roa N.Y., Rodríguez-Pulido A.I. (2013). Occupational exposure to air pollutants: Particulate matter and respiratory symptoms affecting traffic-police in Bogotá. Rev. De Salud Pública.

[8] Karottki D.G., Spilak M., Frederiksen M., Gunnarsen L., Brauner E.V., Kolarik B., Andersen Z.J., Sigsgaard T., Barregard L., Bo S. (2013). An indoor air filtration study in homes of elderly: Cardiovascular and respiratory effects of exposure to particulate matter. Environ. Health.

[9] Karakatsani A., Analitis A., Perifanou D., Ayres J.G., Harrison R.M., Kotronarou A., Kavouras I.G., Pekkanen J., Hämeri K., Kos G.P. (2012). Particulate matter air pollution and respiratory symptoms in individuals having either asthma or chronic obstructive pulmonary disease: A European multicentre panel study. Environ. Health.

[10] Peng R.D., Chang H.H., Bell M.L., Mcdermott A., Zeger S.L., Samet J.M., Dominici F. (2008). Coarse particulate matter air pollution and hospital admissions for cardiovascular and respiratory diseases among Medicare patients. JAMA.

[11] Hoek G., Krishnan R.M., Beelen R., Peters A., Ostro B., Brunekreef B., Kaufman J.D. (2013). Long-term air pollution exposure and cardio-respiratory mortality: A review. Environ. Health.

[12] Koken P.J.M., Piver W.T., Ye F., Anne E., Olsen L.M., Portier C.J. (2003). Temperature, air pollution, and hospitalization for cardiovascular diseases among elderly people in Denver. Environ. Health Perspect..

[13] Barnett A.G., Williams G.M., Schwartz J., Best T.L., Neller A.H., Petroeschevsky A.L., Simpson R.W. (2006). The Effects of Air Pollution on Hospitalizations for Cardiovascular Disease in Elderly People in Australian and New Zealand Cities. Environ. Health Perspect..

[14] Wong T.W., Lau T.S., Yu T.S., Neller A., Wong S.L., Tam W., Pang S.W. (1999). Air pollution and hospital admissions for respiratory and cardiovascular diseases in Hong Kong. Occup. Environ. Med..

[15] Brook R.D., Franklin B., Cascio W., Hong Y., Howard G., Lipsett M., Luepker R., Mittleman M., Samet J., Smith S.C. (2012). Air pollution and cardiovascular disease. Thromb. Res..

[16] Ballester F., Tenías J.M., Pérezhoyos S. (2001). Air pollution and emergency hospital admissions for cardiovascular diseases in Valencia, Spain. J. Epidemiol. Community Health.

[17] Ping W.G., Wei H.U., Jiang T.E., Sheng W.F. (2001). Analysis of the effect of air pollution on the adult’s respiratory health. Environ. Monit. China.

[18] Yin W.J., Peng X.W., Song S.Z. (2012). Air Pollution and the Cerebro Cardio-vascular Diseases Mortality of Population in Guangzhou:a Time-series Analysis. J. Environ. Health.

[19] Yan Y., Bai Z. (2012). Research Advances in Exposure to Ambient Particulate Matter and Health Effects. Asian J. Ecotoxicol..

[20] China National Health and Family Planning Commission (2017). China Health and Family Planning Statistical Yearbook 2017.

[21] Zinola A. (2006). Impact of Exposure Error on the Relationship Between Traffic-Related Air Pollution and Heart Rate Variability (HRV). Epidemiology.

[22] Wesson K., Fann N., Morris M., Fox T., Hubbell B. (2010). A multi–pollutant, risk–based approach to air quality management: Case study for Detroit. Atmos. Pollut. Res..

[23] Andersson K. (2010). Epidemiological Approach to Indoor Air Problems. Indoor Air.

[24] Von K.S., Peters A., Aalto P., Bellander T., Berglind N., D’Ippoliti D., Elosua R., Hörmann A., Kulmala M., Lanki T. (2005). Ambient air pollution is associated with increased risk of hospital cardiac readmissions of myocardial infarction survivors in five European cities. Circulation.

[25] Villeneuve P.J., Chen L., Stieb D., Rowe B.H. (2006). Associations between outdoor air pollution and emergency department visits for stroke in Edmonton, Canada. Eur. J. Epidemiol..

[26] Huang W., Tan J., Kan H., Zhao N., Song W., Song G., Chen G., Jiang L., Jiang C., Chen R. (2016). Visibility, air quality and daily mortality in Shanghai, China. Sci. Total Environ..

[27] Achilleos S., Kioumourtzoglou M.-A., Wu C.-D., Schwartz J.D., Koutrakis P., Papatheodorou S.I. (2017). Acute effects of fine particulate matter constituents on mortality: A systematic review and meta-regression analysis. Environ. Int..

[28] Yang Z., Xu Q., Chen Y., Tsui K.L. (2018). Using Baidu index to nowcast hand-foot-mouth disease in China: A meta learning approach. BMC Infect. Dis..

[29] He G., Chen Y., Chen B., Wang H., Shen L., Liu L., Suolang D., Zhang B., Ju G., Zhang L. (2018). Using the Baidu Search Index to Predict the Incidence of HIV/AIDS in China. Sci. Rep..

[30] Huang X., Zhang L., Ding Y. (2016). The Baidu Index: Uses in predicting tourism flows—A case study of the Forbidden City. Tour. Manag..

[31] Xiong L.F., Zhen F., Wang B., Guang-Liang X.I. (2013). The Research of the Yangtze River Delta Core Area’s City Network Characteristics Based on Baidu Index. Econ. Geogr..

[32] Li Z., Liu T., Zhu G., Lin H., Zhang Y., He J., Deng A., Peng Z., Xiao J., Rutherford S. (2017). Dengue Baidu Search Index data can improve the prediction of local dengue epidemic: A case study in Guangzhou, China. PLoS Negl. Trop. Dis..

[33] Huang X.K., Zhang L.F., Ding Y. (2013). Study on the predictive and relationship between tourist attractions and the Baidu Index: A case study of the Forbidden City. Tour. Trib..

[34] Xie T., Yang Z., Yang S., Wu N., Li L. (2014). Correlation between reported human infection with avian influenza A H7N9 virus and cyber user awareness: What can we learn from digital epidemiology?. Int. J. Infect. Dis..

[35] Liu K., Li L., Tao J., Chen B., Jiang Z., Wang Z., Chen Y., Jiang J., Hua G. (2016). Chinese Public Attention to the Outbreak of Ebola in West Africa: Evidence from the Online Big Data Platform. Int. J. Environ. Res. Public Health.

[36] Yu Z., Zhong S., Wang C., Yang Y., Yao G., Huang Q. (2017). Mapping Comparison and Meteorological Correlation Analysis of the Air Quality Index in Mid-Eastern China. Int. J. Geo-Inf..

[37] Wang Y., Song T., Gao W., Ji D., Wang L., Yao L., Li X. (2016). The Challenge and Opportunity on Preventing and Controlling Air Pollution of Beijing. Bull. Chin. Acad. Sci..

[38] Fu X.Y. (2014). Development and Current Situation of Ambient Air Quality Standard in China. Environ. Sustain. Dev..

[39] Quan K. (2012). Standard revision information—Ministry of Environmental Protection issued GB3095-2012 Environmental Air Quality Standards. China Stand. Rep..

[40] Li Z. (2012). Baidu-index-based Analysis of Regional Network Attention: Taking the Case of Zhenjiang. Libr. Inf. Stud..

[41] Adler J., Parmryd I. (2010). Quantifying colocalization by correlation: The Pearson correlation coefficient is superior to the Mander’s overlap coefficient. Cytom. Part A.

[42] Goldreich Y., Raveh A. (2010). COPLOT Display Technique as an Aid to Climatic Classification. Geogr. Anal..

[43] Brunsdon C. (2001). The comap: Exploring spatial pattern via conditional distributions. Comput. Environ. Urban Syst..

[44] Hastie T., Tibshirani R. (1995). Generalized additive models for medical research. Stat. Methods Med. Res..

[45] Richards R., Hughes L., Gee D., Tomlinson R. (2013). Using generalized additive models for water quality assessments: A case study example from Australia. J. Coastal Res..

[46] Xiang N.M., Sen L.X., Cheng X.Y. (2010). Effects of spatiotemporal and environmental factors on the fishing ground of Trachurus murphyi in Southeast Pacific Ocean based on generalized additive model. Chin. J. Appl. Ecol..

[47] Guisan A., Edwards T.C., Hastie T. (2002). Generalized linear and generalized additive models in studies of species distributions: Setting the scene. Ecol. Model..

[48] Dominici F., Mcdermott A., Zeger S.L., Samet J.M. (2002). On the Use of Generalized Additive Models in Time-Series Studies of Air Pollution and Health. Am. J. Epidemiol..

[49] Schwartz J., Spix C., Touloumi G., Bachã R.L., Barumamdzadeh T., Le T.A., Piekarksi T., Ponce D.L.A., Pãnkã A., Rossi G. (1996). Methodological issues in studies of air pollution and daily counts of deaths or hospital admissions. J. Epidemiol. Community Health.

[50] Ostro B., Broadwin R., Green S., Feng W.-Y., Lipsett M. (2006). Fine particulate air pollution and mortality in nine California counties: Results from CALFINE. Environ. Health Perspect..

[51] Samoli E., Aga E., Touloumi G., Nisiotis K., Forsberg B., Lefranc A., Pekkanen J., Wojtyniak B., Schindler C., Niciu E. (2006). Short-term effects of nitrogen dioxide on mortality: An analysis within the APHEA project. Eur. Respir. J..

[52] Peng R.D., Dominici F., Louis T.A. (2006). Model choice in time series studies of air pollution and mortality. J. R. Stat. Soc. Ser. A (Stat. Soc.).

[53] Core R Team R: A Language and Environment for Statistical Computing; Publisher: R Foundation for Statistical Computing, 2017. https://www.gbif.org/tool/81287/r-a-language-and-environment-for-statistical-computing.

[54] Deng J., Qin B., Wang B. (2015). Quick implementing of generalized additive models using R and its application in bluegreen algal bloom forecasting. Chin. J. Ecol..

[55] Zhang J., Yu K.F. (1998). What’s the Relative Risk?: A Method of Correcting the Odds Ratio in Cohort Studies of Common Outcomes. JAMA.

[56] Luginaah I.N., Fung K.Y., Gorey K.M., Webster G., Wills C. (2005). Association of ambient air pollution with respiratory hospitalization in a government-designated "area of concern": The case of Windsor, Ontario. Environ. Health Perspect..

[57] Cairncross E.K., John J., Zunckel M. (2007). A novel air pollution index based on the relative risk of daily mortality associated with short-term exposure to common air pollutants. Atmos. Environ..

[58] Wong T.W., Tam W.W.S., Yu I.T.S., Lau A.K.H., Pang S.W., Wong A.H.S. (2013). Developing a risk-based air quality health index. Atmos. Environ..

[59] Sicard P., Lesne O., Alexandre N., Mangin A., Collomp R. (2011). Air quality trends and potential health effects—Development of an aggregate risk index. Atmos. Environ..

[60] Sicard P., Talbot C., Lesne O., Mangin A., Alexandre N., Collomp R. (2012). The Aggregate Risk Index: An intuitive tool providing the health risks of air pollution to health care community and public. Atmos. Environ..

[61] Bell M.L., Mcdermott A., Zeger S.L., Samet J.M., Dominici F. (2004). Ozone and short-term mortality in 95 US urban communities, 1987–2000. JAMA.

[62] Ye Y. (2009). A Case-Crossover Study on the Relationship between Air Pollution and Acute Onset of Cardio-Cerebrovascular Disease. Doctoral Dissertation.

[63] Mücke H.G. (2000). Ambient air quality programmes for health impact assessment in the WHO European region. Arhiv Za Higijenu Rada I Toksikologiju.

[64] Künzli N., Kaiser R., Medina S., Studnicka M., Chanel O., Filliger P., Herry M., Horak F., Puybonnieux-Texier V., Quénel P. (2000). Public-health impact of outdoor and traffic-related air pollution: A European assessment. Lancet.

[65] Zhou M., He G., Liu Y., Yin P., Li Y., Kan H., Fan M., Xue A., Fan M. (2015). The associations between ambient air pollution and adult respiratory mortality in 32 major Chinese cities, 2006–2010. Environ. Res..

[66] Chen R., Yin P., Meng X., Liu C., Wang L., Xu X., Ross J.A., Tse L.A., Zhao Z., Kan H. (2017). Fine particulate air pollution and daily mortality. A nationwide analysis in 272 Chinese cities. Am. J. Respir. Crit. Care Med..

[67] Wang L., Liu C., Meng X., Niu Y., Lin Z., Liu Y., Liu J., Qi J., You J., Tse L.A. (2018). Associations between short-term exposure to ambient sulfur dioxide and increased cause-specific mortality in 272 Chinese cities. Environ. Int..

[68] Lai H.-K., Tsang H., Wong C.-M. (2013). Meta-analysis of adverse health effects due to air pollution in Chinese populations. BMC Public Health.

